# Assortativity and the Probability of Epidemic Extinction: A Case Study of Pandemic Influenza A (H1N1-2009)

**DOI:** 10.1155/2011/194507

**Published:** 2010-12-23

**Authors:** Hiroshi Nishiura, Alex R. Cook, Benjamin J. Cowling

**Affiliations:** ^1^PRESTO, Japan Science and Technology Agency, Saitama 332-0012, Japan; ^2^Theoretical Epidemiology, University of Utrecht, Yalelaan 7, 3584 CL Utrecht, The Netherlands; ^3^Department of Statistics and Applied Probability, National University of Singapore, Singapore 117546; ^4^School of Public Health, The University of Hong Kong, Hong Kong Special Administrative Region, Hong Kong

## Abstract

Unlike local transmission of pandemic influenza A (H1N1-2009), which was frequently driven by school children, most cases identified in long-distance intranational and international travelers have been adults. The present study examines the relationship between the probability of temporary extinction and the age-dependent next-generation matrix, focusing on the impact of assortativity. Preferred mixing captures as a good approximation the assortativity of a heterogeneously mixing population. We show that the contribution of a nonmaintenance host (i.e., a host type which cannot sustain transmission on its own) to the risk of a major epidemic is greatly diminished as mixing patterns become more assortative, and in such a scenario, a higher proportion of non-maintenance hosts among index cases elevates the probability of extinction. Despite the presence of various other epidemiological factors that undoubtedly influenced the delay between first importations and the subsequent epidemic, these results suggest that the dominance of adults among imported cases represents one of the possible factors explaining the delays in geographic spread observed during the recent pandemic.

## 1. Introduction

Since it was first identified in early 2009, a novel strain of influenza A (H1N1-2009) has caused a global pandemic. Although the rapid international spread created various epidemiological challenges, such as quantifying the strain's transmission potential and virulence during the very early stages of the pandemic [1, 2], many key insights have been obtained to date [3]. Prior to the pandemic, the importance of contact networks in elucidating the epidemiological dynamics of infectious diseases has been emphasized with applications to severe acute respiratory syndrome (SARS), sexually transmitted infections and other directly transmitted diseases [4, 5]. The age specificity in the transmission of the H1N1-2009 indicates the relevance of contact heterogeneity [6–9]. Although the differential attack rates in different age groups by H1N1-2009 have multiple explanatory factors, including age-specific susceptibility and pre-existing immunity [10–12], age-dependent contact is also thought to be associated with a higher susceptibility to infection and greater infectiousness once infected in children [13]. The consequences of this are that community-wide epidemics have been frequently driven by school outbreaks [7], while attack rates of H1N1-2009 were highest among school-age children in various parts of the world [8, 11]. A network model was used to describe the temporal variations in the age-specific composition of cases during the course of the pandemic and found that attack rates for a novel strain of influenza tend to be initially biased towards children and then shift towards adults [14].

A parsimonious simplification of the complexity of an age-structured contact network can be obtained by approximating the network by an appropriately quantified age-dependent next-generation matrix. This is accomplished by using the next-generation matrix, the square matrix with generic entry *R*_*ij*_, the average number of secondary cases in age-group *i* generated by a single primary case in age-group *j* in a fully susceptible population. Discretizing chronological age into a small number of age groups, the matrix, **K** = {*R*_*ij*_}, for H1N1-2009 has been quantified using age-stratified epidemic data [2, 6, 7, 15]. The age-dependent **K** has two important properties in understanding epidemiological dynamics. First, the dominant eigenvalue of **K** corresponds to the basic reproduction number, *R*_0_ [16], which frequently yields a threshold condition, that is, a major epidemic is possible if and only if *R*_0_ > 1. Second, the final proportion of hosts of type *i* that become infected, *z*_*i*_, is given by the solution to 1 − *z*_*i*_ = exp (−∑_*j*_ *R*_*ij*_ *z*_*j*_) [17]. One important use of *R*_*ij*_ is to the development of optimal vaccination strategies before a pandemic [18, 19] and during a pandemic [20].

The present study investigates the relationship between the age-dependent next-generation matrix, **K**, and the invasion of a novel virus into a large population, which has not been well clarified to date. Whereas this subject has been partly explored via percolation theory [5], it is important from an epidemiological perspective to address this using a simpler model that can more readily be fitted to observational data from the outbreak in question. More specifically, we examine the impact of assortativity, that is, preferential mixing of different host types (here, age), on the probability of epidemic extinction, because age-dependent human contact networks have been shown to be highly assortative in contact surveys [21, 22]. Such an assortative network is known to allow disease percolation more easily than disassortative ones [23], echoed by the finding that increasing the preferential mixing component of simpler models such as ours tends to allow an epidemic to grow more easily [24].

In addition to these issues, the present study investigates the role of the age of cases importing infection to a local area by long-distance travel either intranationally or internationally on the resulting growth of a local epidemic. As a practical example, the age-dependent transmission of the H1N1-2009 pandemic is considered, and we first present our study motivations in the next section.

## 2. Materials and Methods

### 2.1. Study Motivation

**Figure 1:**
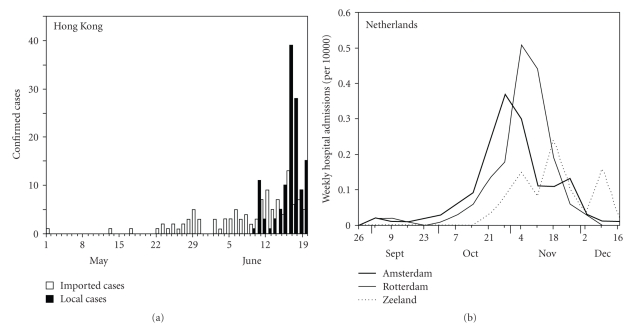
Epidemiology of the pandemic influenza A (H1N1-2009) in Hong Kong and the Netherlands. (a) Introduction of imported confirmed cases in Hong Kong followed by an increase in local (indigenous) confirmed cases from May–June, 2009 [25]. (b) The hospitalization rates of the influenza A (H1N1-2009) in Amsterdam, Rotterdam and Zeeland from August–December, 2009 [26]. Weekly numbers of hospitalizations are divided by the population size in each locality.

Figure 1(a) shows the time delay from the introduction of first imported case on 1 May 2009 to the subsequent increase in local transmission in Hong Kong [25]. Despite the number of imported cases, it took 39 days to observe the first locally acquired case. The interpretation of Figure 1(a) is affected by several factors, such as case ascertainment, ecological factors such as seasonality, and disease control [27], but as with many other countries exponential growth in the local epidemic did not start for some time after the first imported case. Hong Kong instigated particularly stringent quarantine measures, but a recent study comparing the time delay in local transmission between countries with and without entry screening has shown that the entry screening measures were not associated with a substantial delay in the start of local transmission [28].

Figure 1(b) shows weekly hospitalization rates due to H1N1-2009 in three coastal areas in the Netherlands [26]. A surge in hospitalizations is first seen in Amsterdam followed by Rotterdam. The peak hospitalization rate in Zeeland occurs three weeks later than that in Amsterdam. There may be various interpretations for the delay before exponential growth, and, in particular, the spatial heterogeneity in Figure 1(b) is likely to have been associated with differing inflows of infected individuals and intrinsically differing patterns of spread within each region. Despite the presence of various possible factors explaining Figure 1(b), it is clear that the spatio-temporal dynamics are not synchronized even in this geographically limited country, and thus, Figure 1(b) at least indicates that stochastic effects may not have been insignificant for the intranational spread. A similar substantial delay in interregional spread has also been seen in the results of seroepidemiological study in England [29].

Both Figures 1(a) and 1(b) indicate a delay in causing international or interregional spread, but from a sufficiently high number of homogeneous index cases that repeated stochastic extinction is unlikely as an explanation. A more plausible reason is the contrast in age distributions between local and imported cases: whereas imported cases have been predominantly adults [30], local transmissions are frequently driven by school children. That is, adults were more likely to travel than children, and those aged 25 years and older accounted for more than half of the imported cases in Japan [30]. Similarly, adults may also more likely be the source of spread within a country, especially as the movement distance becomes longer. However, adults are less likely to cause secondary transmissions than children in a local setting [2, 6, 13, 15], making it critically important to understand the differential probability of extinction of the infection tree emerging from a typical child index and a typical adult index case. Because assortativity regulates the frequencies of within- and between-group transmissions, examining the effects of assortativity provides a natural avenue for assessing this. Accordingly, in this paper we use a simple stochastic model to clarify the different roles of children and adults in causing a major epidemic and its relevance to assortativity.

### 2.2. A Model for Clade Extinction

 We employ a multitype branching process to approximate the probability of extinction of the clade of infection emanating from a single index case [31]. Consider a large population which is fully susceptible, and let there be two subpopulations, that is, children and adults. For simplicity, we ignore pre-existing immunity among adults. Throughout this paper, we label children as type 1 and adults as type 2. Let *γ*_*i*_ (*i* = 1,2) be the recovery rate of infectious individuals of type *i* and *β*_*ij*_(1 ≤ *i*, *j* ≤ 2) be the birth rate (i.e., the rate of new infection) of type *i* infected individuals caused by a single type *j* infected individual during the initial stage of an epidemic. We consider the case when a small number of *a*_*i*_ infected individuals of type *i* invades a fully susceptible large population. Given the large and (assumed) fully susceptible population, and the small initial number of infectives, depletion of the susceptible stock can be ignored and the initial stages of the outbreak interpreted as a multivariate birth-and-death process [32]. For mathematical convenience, we assume that the generation time is exponentially distributed, and thus, *R*_*ij*_ = *β*_*ij*_/*γ*_*j*_. This assumption is common to many compartmental models, although its realism is dubious. The proposed approach considers a linearized system for the early epidemic period with a crude approximation of host types, but the similar approach of mapping next generation with the use of a square matrix can be employed for explicit network models [33]. In addition to the aforementioned assumptions, we assume that the age-specificity of *R*_*ij*_ is fully attributable to the infection rate *β*_*ij*_, and thus the infectious period *γ*_*j*_ is assumed to be a constant *γ*, independent of host type. Consequently, if we further ignore the age-specific susceptibility and infectiousness, *R*_*ij*_ is determined only by the frequency of contact within- and between-age groups. 

 Letting the random vector **X**_*n*_ = (*X*_1*n*_, *X*_2*n*_) represent the number of child and adult infected individuals in the population in the *n*th generation, we consider the process {**X**_*n*_} as a multitype branching process. Assuming that an individual of type *j* has probability *p*_*j*_(**x**) of infecting, in the next generation, *x*_1_ children and *x*_2_ adults, we define the probability generating function as

(1) $$F_{j}\left(s_{1},s_{2}\right)=\underset{x}{\sum }p_{j}\left(x_{1},x_{2}\right)s_{1}^{x_{1}}s_{2}^{x_{2}},\;j=1,2.$$

Following foregoing studies [32, 34], the generating function *F*_*j*_(**s**) with an exponentially distributed generation time is known to be given by

(2) $$F_{j}\left(s\right)=\frac{\gamma_{j}}{\gamma_{j}+\sum_{k=1}^{2}\beta_{kj}\left(1-s_{k}\right)}$$

for *j* = 1,2. Since *γ*_*j*_ is assumed to be independent of host type *j*, *R*_*ij*_ = *β*_*ij*_/*γ*, (2) simplifies to

(3) $$F_{j}\left(s\right)=\frac{1}{1+R_{1j}\left(1-s_{1}\right)+R_{2j}\left(1-s_{2}\right)}.$$

The clade of infections, {**X**_*n*_}, emanating from the initial index cases becomes extinct with probability 1 if and only if the dominant eigenvalue of **K** is less than or equal to unity, that is, *ρ*(**K**) ≤ 1 [34].

 Let *π*_*i*_ be the probability of extinction given that a single infected individual of type *i* is introduced to the population. The extinction probability is the nonnegative root of the equations

(4) $$\pi_{j}=F_{j}\left(\pi \right),\;j=1,2.$$

As is standard in branching process models, each of the secondary cases of type *i* generated by a primary case becomes an ancestor of an independent subprocess (which restarts with a type *i* individual) behaving identically among the same type *i* [31, 35]. Because of this multiplicative nature, we have the probability of extinction

(5) $$p\left(a\right)=\overset{2}{\underset{j=1}{\prod }}\left\{\pi_{j}^{a_{j}}\right\}$$

of the entire clade with initial vector **a** = (*a*_1_, *a*_2_). 

In the two-host population, that is, a population consisting of children and adults, the probabilities of extinction given a single child or adult infected individual, *π*_1_ and *π*_2_, satisfy

(6) $$\begin{array}{c} \pi_{1}=\frac{1}{1+R_{11}\left(1-\pi_{1}\right)+R_{21}\left(1-\pi_{2}\right)},\\ \pi_{2}=\frac{1}{1+R_{12}\left(1-\pi_{1}\right)+R_{22}\left(1-\pi_{2}\right)}. \end{array}$$

In other words, given that the next-generation matrix *R*_*ij*_ is known, the problem of calculating the probability of extinction given a certain number of infected individuals of host *i* and *j* in the zero generation is replaced by the problem of solving two quadratic equations with two unknown parameters. There are four possible combinations of the solutions for (6) including complex numbers, but we iteratively find the only nonnegative real numbers in the range of 0 ≤ *π*_1_, *π*_2_ ≤ 1 (see [31, page 18]), except for a combination (*π*_1_, *π*_2_) = (1,1).

### 2.3. Quantitative Illustrations

The probability of extinction is investigated for the following three different scenarios. First, to gain an overview of the extinction probabilities *π*_1_ and *π*_2_ for the H1N1-2009, (6) are solved using published estimates of **K** from Mexico [6] and Japan [2]. Approximating the original **K** into a two-host population, we use

(7) $$\begin{array}{c} K_{a}=\left(\begin{array}{cc} 1.41 & 0.34\\ 0.35 & 0.87 \end{array}\right) \end{array}$$

for Mexico, and

(8) $$\begin{array}{c} K_{b}=\left(\begin{array}{cc} 1.14 & 0.25\\ 0.21 & 0.45 \end{array}\right) \end{array}$$

for Japan. The originally estimated dominant eigenvalues are 1.58 and 1.22, respectively. It should be noted that the child group in Mexico is assumed to be up to age 14 years while that in Japan is up to age 19 years. Assuming that the reproduction number, *R* possibly ranges from 1.2–1.6 [2, 36], we rescale the next-generation matrices by

(9) $$K_{q}^{\prime }=\frac{R}{\rho \left(K_{q}\right)}K_{q},\;q=a,b,$$

where *R* is the reproduction number to be examined.

**Figure 2:**
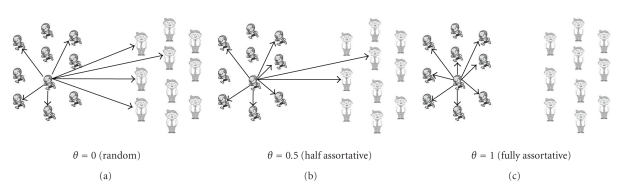
Preferential mixing given a single child index case. The hypothetical population consists of 10 children (left) and 10 adults (right) with equal susceptibility and infectiousness. We consider an introduction of a single child index case who has a potential to cause 8 secondary transmissions. Panels (a)–(c) illustrate contacts generated by the child index case with different *θ*, proportion of within-group contacts, being 0, 0.5 and 1.0, respectively. With *θ* = 0 (i.e., random mixing), four edges extend to child susceptibles and the other four to adult susceptibles. Nevertheless, with *θ* = 0.5, additional two edges are reserved for within-child mixing and only the remaining two are connected to adult susceptibles. With *θ* = 1 (i.e., fully assortative mixing), all edges are connected with child susceptibles.

 Second, **K**_*a*_ in Mexico is further examined in relation to the assortativity. The element *R*_*ij*_ of **K**_*a*_ has been parameterized as

(10) $$R_{ij}\sim \{\begin{array}{cc} \left(1-\theta \right)\alpha_{i}\beta_{j}n_{i}, & \text{for}\;i\neq j,\\ \theta \alpha_{i}\beta_{j}+\left(1-\theta \right)\alpha_{i}\beta_{j}n_{i}, & \text{for}\;i=j, \end{array}$$

where *n*_*i*_ is the relative size of the subpopulation *i* (i.e., *n*_1_ + *n*_2_ = 1). *α*_*j*_ and *β*_*j*_ are originally described as the age-specific susceptibility and infectiousness [6], and these can also be regarded as the so-called proportionate mixing components. The biological interpretation of proportionate mixing is that irrespective of its own type, an individual can acquire infection from any given individual (i.e., the secondary transmission from host *j* to *i* is determined by host *j*). Introduction of the most important parameter in the present study, *θ* is classically referred to as “preferred” or “preferential” mixing [37, 38]. Although the original definition of the term preferred mixing has a broader meaning, *θ* in (10) represents the proportion of contacts reserved for within-group mixing, and (1 − *θ*) represents the proportion of contacts subject to proportionate mixing. If *θ* = 1, the mixing is referred to as fully assortative (Figure 2). If *θ* = 0, the mixing corresponds to random mixing (though it should be noted that the mixing matrix still includes a proportionate mixing component). An empirical estimate of *θ* from Mexico is 0.50, although the 95% confidence interval is broad: 0–0.72 [6]. Therefore, we examine the sensitivity of the probabilities of extinction, *π*_1_ and *π*_2_, to different *θ* in the range of 0-1 and *R* in the range of 1.2–1.6. Other parameters are fixed at *n*_1_ = 0.32, *α*_1_ = 2.06, *α*_2_ = *β*_1_ = *β*_2_ = 1 [6].

 Third, to clarify the practical implications of the predomination of adults among travelers, we examine the sensitivity of the probability of extinction to the proportion of adult travelers over various *θ* and *R*. Specifically, we consider the probability of extinction given a small importation of ten cases in the zero generation independently entering a large susceptible population at their infection-age 0 (i.e., immediately after their own infections: for simplicity, we ignore the infection-age distribution of imported cases at the time of invasion in the present study, because its realistic incorporation enforces us to account for the epidemic dynamics in exporting countries and thus, the exporting country and travel distance for each imported case would be required [39]). Among the 10 cases, we vary the number of adult cases from 0 to 10, and examine the probability of extinction given by (5).

## 3. Results

**Figure 3:**
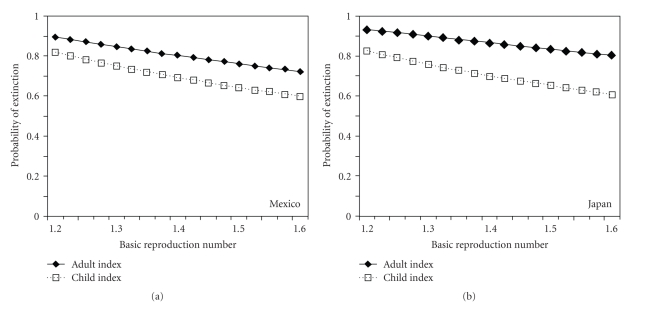
Probability of extinction given an introduction of single child or adult index case. The probabilities of extinction are calculated, assuming that a single child or adult index case is introduced into a fully susceptible large population. The probability approximately accounts for heterogeneous transmission among and between child and adult populations. The estimates of the next-generation matrices are extracted from A. Fraser et al. [6] based on an analysis in Mexico and B. Nishiura et al. [2] in Japan. The basic reproduction numbers in the original studies in Mexico and Japan are estimated at *R*_0_ = 1.58 and 1.22, respectively, and both panels rescales the next-generation matrix by multiplying each entry with *R*/*R*_0_ where *R* measures the horizontal axis.

Figure 3 shows the probabilities of extinction, *π*_1_ and *π*_2_, using published estimates of **K**_*a*_ and **K**_*b*_ in (7) and (8). In both panels, using estimates from Mexico and Japan, *π*_2_, the probability of extinction given a single adult case, always appeared to be higher than *π*_1_, and thus the clade of infections resulting from the introduction of an adult index case is more likely to be self-limiting than from a child index case. The estimates of *π*_1_ and *π*_2_ using the published estimates of *R*_0_ were 61 and 73%, respectively, for Mexico (with *R*_0_ = 1.58) and 81 and 93%, respectively, for Japan (with *R*_0_ = 1.22), indicating that the reproduction number *R* in the range of 1.2–1.6 is not far from the critical level and the impact of variations in *R*_0_ on epidemic extinction is large. The reader should note the crudeness of the dichotomization of the population into two subpopulations, and that incorporating more detailed network structure (e.g., by dividing the population into many more types of host) tends to yield higher probability of extinction [2, 5]. Moreover, whereas the present study assumes an exponentially distributed generation time, a more realistic depiction, for example, gamma-distributed generation time, tends to capture overdispersion of the offspring distribution more appropriately [40, 41], and thus, again yields a higher probability of extinction than is shown herein.

**Figure 4:**
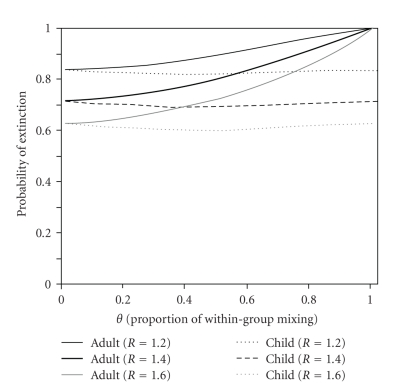
Assortativity and the probability of extinction. The probability of extinction in a fully susceptible population given a single adult or child index case is measured as a function of *θ*, the proportion of within-group mixing. The next-generation matrix, parameterized by Fraser et al. [6] with the dominant eigenvalue *R*_0_ = 1.58, is rescaled by multiplying each entry with *R*/*R*_0_ where *R* is set to be 1.2, 1.4 and 1.6, respectively.

Figure 4 examines the probabilities of extinction, *π*_1_ and *π*_2_, as a function of *θ*, the proportion of within-group mixing and *R*. As expected from the randomly mixing interpretation, *π*_1_ and *π*_2_ were equal to 1/*R* with *θ* = 0. However, for populations with more within-group mixing, clades from adults were more likely to go extinct, reaching 100% with *θ* = 1. This is attributable to the next-generation matrices (7) and (8) involving the typical reservoir dynamics [42]: children act as a maintenance host (*R*_11_ > 1), among whom transmission can be maintained by themselves, while adults constitute a nonmaintenance host group (*R*_22_ < 1), and thus, with little relative mixing between the two groups, an adult index case would never lead to a major epidemic. The probability of extinction, *π*_1_, given a single child index case, reached a minimum with *θ* in the range of 0.4-0.5, although this probability was not very sensitive to *θ*. Such *θ* may lead to a “well-mixed” population, thereby allowing the child index case to involve both child and adult secondary cases effectively.

**Figure 5:**
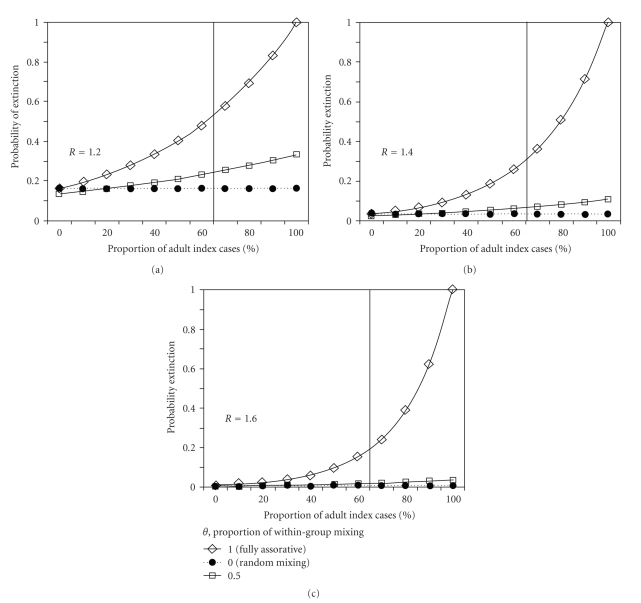
The impact of the age specificity of index cases on the probability of extinction. All panels, (a)–(c), examine the sensitivity of the probability of extinction in a fully susceptible large population given 10 index cases with *R* = 1.2, 1.4 and 1.6, respectively, with different *θ*, the proportion of within-group mixing and the proportion of adult index cases. Vertical grey bold line represents the empirically observed proportion of adult imported cases in Japan (i.e., 63.4% were aged <15 years). The horizontal axis measures the proportion of adult index cases among the total number of 10 index cases. The next-generation matrix, parameterized by Fraser et al. [6] with the dominant eigenvalue *R*_0_ = 1.58, is rescaled by multiplying each entry with *R*/*R*_0_.

Figure 5 examines the probability of extinction given 10 index cases as a function of *θ* and the proportion of adult index cases. With random mixing, the probability of extinction given a single index case is (100/*R*)% (e.g., 71% with *R* = 1.4). The extinction probability given 10 index cases in the randomly mixing population was independent of the proportion of adults, (1/*R*)^10^ = 0.035 (with *R* = 1.4), indicating that a major epidemic is almost unavoidable without any intervention. However, as assortativity increased, the increase in the proportion of adult index cases promoted extinction. The vertical reference line of 63% indicates the empirically observed proportion of adults (i.e., those aged ≥15 years in (7) as defined by [6]) among all imported cases in Japan [30]. At around that proportion, the probability of extinction was estimated to be 1%–58% over the full range of *θ* from 0 to 1, for 10 index cases. Given 10 index cases with *θ* = 1, the results were independent of the proportion of adult index cases, because *a*_1_ + *a*_2_ = 10 and *π*_2_ = 1 (for *θ* = 1), whatever the number of adults, *a*_2_, the extinction probability is

(11) $$p\left(a_{1},a_{2}\right)=\pi_{1}^{a_{1}}\pi_{2}^{a_{2}}=\pi_{1}^{a_{1}}.$$

That is, as mixing becomes more and more assortative, the contribution of the initial number of adults, *a*_2_ to the risk of a major epidemic becomes less important, and moreover, an increase in the proportion of adults indirectly reduces *a*_1_, leading to an increase in the probability of extinction. Even when we divide the entire population into many more subpopulations, this argument holds as long as the host type of interest *i* is incapable of maintaining disease by itself, that is, when the host-specific reproduction number, *R*_*ii*_ < 1 [42]. Of course, if *a*_2_ = 10, *p*(0,10) = 1 for *θ* = 1.

## 4. Discussion

The present study investigated the relationship between the next-generation matrix and the probability of extinction, employing a simple model that may be viewed as an approximation to a full network model. The modelled heterogeneous mixing accounted for assortativity via an assumption of preferred mixing, and the probability of extinction was derived from a multidimentional branching process model. As a practical example, the age dependency in the transmission of pandemic influenza A (H1N1-2009) was considered, dividing the population into children and adults. Through quantitative illustrations, it has been shown that the probability of extinction given an adult index case increases with *θ*, at least for diseases with similar transmissibility as influenza. Although this exercise employed several simplifying assumptions, a formal hypothesis can be developed for explaining a slow interregional and international spread of the H1N1-2009 even in today's highly mobile world population. That is, whereas empirically observed delays in local transmission can be influenced by a large number of factors including pre-existing immunity, public health interventions and seasonality, the dominance of adults among travelers is one possible explanation for the high probability of extinction, and may play an important role in describing the underlying reason (Figure 1). Since the present study adopted three simplifying assumptions (i.e., (1) the crude dichotomization of hosts into two different types, (2) the adoption of exponentially distributed generation times, and (3) ignorance of infection-age among imported cases), the probability of extinction is likely to have been underestimated. The extinction probabilities become higher with more precise network structure (e.g., due to localized burnout of susceptible individuals) and more detailed natural history of infection [5, 40, 41].

Three practical implications are drawn from our exercise. First, the importance of assortativity in appropriately capturing the probability of extinction highlights a critical need to account for this aspect when quantifying the next-generation matrix in an approximately modelled heterogeneous population. Whenever the statistical inference of the next-generation matrix is made for directly transmitted diseases, the estimation framework should ideally account for assortative mixing. Whereas the social contact survey revealed that the age-dependent contact pattern is highly assortative [20, 21], the definition of a contact can be too broad to be practical for all diseases, and more realistic incorporation of assortative mixing and its precise estimation should be the subject of future studies.

Second, as was highlighted with an application, accounting for the age specificity in the surveillance of international and interregional mobility patterns and its use for statistical inference of epidemic dynamics are of utmost importance. For example, global airline transportation is one of the most well-studied networks, and this has been analyzed for H1N1-2009 [43], but a full description of global dynamics should better account for age-specific travel patterns. In addition, whereas imported cases from Mexico have been utilized to make statistical inference (e.g., spatial backcalculation) of the incidence in Mexico [6, 44, 45], the present study emphasizes a critical need to examine age-specificity in relevant frameworks, so that ultimately, the global dynamics can be described by a multihost metapopulation model [46, 47].

Third, as a disease control implication, although adults dominate imported cases, it should be remembered that the more important target host is still children. If stringent border control measures, for example, travel reduction and movement restrictions among all incoming passengers [48], are adopted as containment strategies against a highly virulent novel virus, the target host to promote radical reductions in travel-induced illness would be children, at least for diseases with a similar next-generation matrix to that of the recent pandemic.

Although the role of heterogeneously mixing population in the spread of infectious diseases has been examined using stochastic modelling approaches, past studies tended to focus on final epidemic size and its relevance to disease control policy [49, 50]. Moreover, a limited number of studies examining the probability of extinction took an average of the probabilities over different types of host (e.g., by weighting the relative population size to the type specific probability of extinction) [24, 49]. The present study emphasized the importance of capturing type-specificity of index cases in estimating the probability of extinction and examining the impact of assortativity on extinction. In conclusion, we believe our simple exercise successfully illustrated the diminished role of nonmaintenance hosts in causing a major epidemic when assortativity is high, indicating a critical need to capture the assortativity in modelling the initial invasion of an epidemic disease.

## 5. Conclusions

Unlike local transmission of the H1N1-2009 which was frequently driven by school children, imported cases were predominantly adults. This study examined the relationship between the age-dependent next-generation matrix and the probability of extinction, focusing on the role of nonmaintenance hosts and the impact of assortativity on the epidemic extinction. The preferred mixing assumption captures assortativity in a much simpler way than full contact network models, allowing analysis in place of Monte Carlo calculations. The contribution of nonmaintenance hosts to the risk of a major epidemic is diminished as the mixing pattern becomes more assortative, so that an increase in the proportion of nonmaintenance hosts among index cases increases the probability of extinction, if temporary in the face of repeat importations. These results helped us to formulate a hypothesis that the dominance of adults in imported cases was one of the possible causes of observing substantial delay in interregional and international spread of the 2009 influenza pandemic. The importance of capturing the assortativity in estimating the next-generation matrix was highlighted.
